# Preservative Potential of *Anethum graveolens* Essential Oil on Fish Fillet Quality and Shelf Life During Refrigerated Storage

**DOI:** 10.3390/foods14091591

**Published:** 2025-04-30

**Authors:** Aya Tayel, Faten S. Hassanin, Shimaa N. Edris, Ahmed Hamad, Islam I. Sabeq

**Affiliations:** Department of Food Hygiene and Control, Faculty of Veterinary Medicine, Benha University, Benha 13511, Egypt; aya.magdy@fvtm.bu.edu.eg (A.T.); faten.mohamed@fvtm.bu.edu.eg (F.S.H.); shimaa.edrees@fvtm.bu.edu.eg (S.N.E.); ahmed.alhussaini@fvtm.bu.edu.eg (A.H.)

**Keywords:** fish fillet, dill essential oil, food safety, food quality, natural preservative, quality preservation

## Abstract

This study estimated the preservative potential of Dill essential oil (DEO, *Anethum graveolens*) in terms of the quality and shelf life of *Pangasius bocourti* (basa fish) fillets during cold storage. GC-MS analysis of DEO’s chemical composition identified monoterpenes, including α-phellandrene (21.81%), d-limonene (18.54%), carvone (17.42%), and Dill ether (14.82%). DEO showed concentration-dependent antioxidant properties in the DPPH assay, with an IC50 of 48.3 ± 0.9 µg/mL (mean ± SE). Its antibacterial efficacy against various foodborne pathogens was evaluated using the resazurin turbidimetric microdilution method. Fish fillets were treated with DEO at 200, 2000, and 4000 ppm, and compared to the untreated control and 200 ppm butylhydroxytoluene (BHT)-treated groups. Physicochemical parameters, microbial growth, and sensory characteristics were assessed over a 15-day period at 2.5 °C ± 0.5 °C. Higher concentrations of DEO effectively preserved the pH, water-holding capacity, and color stability of the fillets. Microbial analysis showed that DEO, particularly at 4000 ppm, significantly inhibited the growth of aerobic bacteria, lactic acid bacteria, coliforms, and staphylococci compared with the control. Sensory evaluation revealed that DEO treatment, especially at 4000 ppm, maintained the odor, color, texture, and overall acceptability of fish fillets throughout storage. These results suggest that *Anethum graveolens* L. essential oil can serve as an effective natural preservative to enhance the quality and prolong the shelf life of refrigerated fish fillets.

## 1. Introduction

Recognized for their health benefits, fish and seafood products are crucial components of human nutrition, supplying at least 20% of the protein consumption for one-third of the global population. The reliance on these food sources is particularly pronounced in developing nations [1]. Fish has been demonstrated to play a crucial role in human nutrition, offering significant advantages for both food security and addressing malnutrition and micronutrient deficiencies in less-developed nations. The value of incorporating fish into diets has been well established, particularly in its ability to combat nutritional challenges faced by developing countries [2]. Fish also has beneficial characteristics, including nutrient-dense protein content and unique flavor. Fish has a clear advantage over other animal and plant products because of the vast diversity of species and wide range of prices. This variety makes fish accessible to individuals across all economic levels, including the high-, middle-, and low-income groups. Consequently, fish has become one of the most widely consumed sources of animal protein [1]. Seafood, especially fish, is highly susceptible to spoilage owing to enzymatic and microbiological degradation. This rapid deterioration can lead to waste with fish product losses potentially reaching up to 40% [3]. Fish and other seafood are particularly prone to decay caused by enzymes and microorganisms. This quick decomposition can result in significant waste, with potential losses of fish products as high as 40% [4]. Fish fillets rapidly deteriorate in nutritional quality, even when stored in refrigeration, due to various processes, including enzymatic browning, non-enzymatic lipid oxidation, and protein breakdown. This deterioration is one of the adverse effects of spoilage and can lead to both health-related and economic issues [5]. Chilling and freezing are the conventional methods for preserving fish fillets. While chilled fish can retain a high sensory quality that consumers find acceptable, it faces potential microbial safety issues due to the temperature range in which it is stored. This is because psychotropic pathogens can proliferate without noticeably affecting the sensory characteristics of the fish [6]. Although freezing can extend the shelf life of fish and fish products, it may lead to changes in texture. This occurs because of the formation of ice crystals and denaturation of proteins, resulting in increased dryness and toughness. These effects are more pronounced in lean fish species compared to fatty or semi-fatty varieties [7].

The majority of preservatives used in meat products, especially in seafood, rely on chemical additives to prolong shelf life and improve sensory appeal [8]. Among the chemical preservatives used in seafood, butylated hydroxytoluene (BHT) stands out as the most frequently utilized option [9]. The utilization of BHT is attributed to its potent antioxidant properties and antimicrobial effects [8]. Nevertheless, BHT has been shown to pose risks to public health including its potential to cause toxic and cancer-inducing effects [10]. Due to these detrimental effects on both the sensory qualities of fresh seafood and consumer health, food safety authorities and certain nations have banned the use of these substances in meat production [10]. Furthermore, both consumers and professionals in the field have recognized the urgent importance of investigating and implementing “bio-preservatives” [11]. EOs and plant extracts have been described in [12].

Recent research has shown that plant essential oils contain antimicrobial properties that can reduce food spoilage bacteria both in vitro and in vivo [13]. Furthermore, the broad antimicrobial properties of essential oils may contribute to preserving seafood freshness, an aspect highly valued by consumers [14]. Moreover, the wide-ranging antimicrobial effects of essential oil could help maintain the freshness of seafood, a quality highly prized by customers [15].

The antioxidant activity of essential oils is mainly associated with the presence of oxygenated monoterpenes and other volatile bioactive compounds that can act as radical scavengers or metal chelators. Additionally, the presence of unpaired electrons allows phenolic compounds to form complexes with metal ions and oxygen, which contributes to the prevention of lipid oxidation [16]. The use of essential oils as antimicrobial agents offers two key advantages: their natural origin makes them generally safer for consumers compared to synthetic chemicals, and they have a reduced likelihood of promoting resistance to dangerous microorganisms. These benefits make essential oils an attractive alternative to conventional antimicrobial substances [17].

One of the annual plants is Dill (*Anethum graveolens* L.), which contains essential oil and belongs to the *Umbelliferae* family. Dill contains compounds such as essential oils, fatty acids, mineral elements (Mn, Ca, Na, Cu, Mg, Fe, K, and phosphorus), vitamins, carbohydrates, proteins, fiber, flavonoids, carotenoids, and phenolic compounds [18]. The primary constituents of DEO, including d-carvone, d-limonene, and α-phellandrene, are responsible for its antibacterial and antioxidant properties [19]. These compounds can protect lipids from oxidation by scavenging reactive oxygen species and free radicals, contributing to fish spoilage [19].

The antimicrobial and antioxidant Dill components enhance the shelf life, sensory, and physicochemical properties of Atlantic bonito fish using Dill leaves [20], fresh fish (*Cyprinus carpio*) meat quality [12], minced meat using Dill EO [21], beef burger using Dill EO [8], refrigerated storage beef using Dill EO [22], and cherry tomatoes using Dill EO [23].

These potent antibacterial qualities make Dill (*Anethum graveolens* L.) essential oil (DEO) a good candidate for prolonging the shelf life of fish products and could prevent the proliferation of foodborne pathogens. To date, few studies have assessed the preservation characteristics of *A. graveolens* EO in fish fillets. Therefore, the current study was conducted to assess the preservative effects of *Anethum graveolens* EO on fillets of Basa fish (*Pangasius bocourti*).

## 2. Material and Methods

### 2.1. Ethical Approval

The research protocol received approval from the Research Ethics Care and Use Committee at Banha University’s Faculty of Veterinary Medicine, with the reference number BUFVTM 18-08-24.

### 2.2. Dill Essential Oil Preparation

At the beginning of the vegetative period, fresh Dill was acquired from a local market. The hydrodistillation process involved placing 50 g of powdered Dill and 750 mL of distilled water into a glass Clevenger apparatus. Essential oil extraction proceeded for 3 h at approximately 100 °C at a distillation rate of 1 mL/min. The resulting essential oil was then collected in pre-weighed vials, using a balance with 0.0001 precision, and stored at a temperature of 4 °C [24].

### 2.3. Determination of the Chemical Compounds of Dill Essential Oil

#### Gas Chromatography–Mass Spectrometry (GC-MS) Analysis

A Trace GC-TSQ mass spectrometer (Thermo Scientific, Austin, TX, USA) equipped with a direct capillary column TG–5MS (30 m × 0.25 mm × 0.25 µm film thickness) was utilized to analyze the chemical composition of the samples. The column oven temperature was initially set at 50 °C, then raised by 5 °C/min to 250 °C and maintained for 2 min. It was further increased to 300 °C at a rate of 30 °C/min and held for 2 min. The injector and MS transfer line temperatures were maintained at 270 °C and 260 °C, respectively. Helium served as the carrier gas, flowing at a constant rate of 1 mL/min. With a solvent delay of 4 min, diluted samples of 1 µL were automatically injected using an Autosampler AS1300 coupled with GC in split mode (Thermo Scientific, Austin, TX, USA). EI mass spectra were gathered in full scan mode at 70 eV ionization voltages across the *m*/*z* range of 50–650. The ion source temperature was configured at 200 °C. Component identification was achieved by comparing their mass spectra with those found in WILEY 09 and NIST 14 mass spectral databases [25].

### 2.4. Dill Oil Antioxidant Activity

The extract’s antioxidant properties were evaluated at Al-Azhar University’s Regional Center for Mycology and Biotechnology (RCMB) using the 2,2-diphenyle-1-picrylhydrazyl (DPPH) free radical scavenging assay. The test was conducted three times, and the mean values were used. The method for assessing DPPH radical scavenging activity followed the protocol outlined by Ang et al. (2015) [26] with some modifications. In summary, a fresh methanol solution (0.004% *w*/*v*) of 2,2-diphenyl-1-picrylhydrazyl (DPPH) radical was created and kept in dark conditions at 10 °C. The test compound was also dissolved in methanol. A 40 uL portion of this solution was introduced to 3 ml of the DPPH solution. A UV–visible spectrophotometer (Milton Roy, Spectronic 1201, Houston, TX, USA) was used to immediately record absorbance measurements. The decline in absorbance at 515 nm was continuously monitored, with data recorded every minute until stabilization occurred (16 min). The absorbance of DPPH radical without antioxidant (control) and the reference compound ascorbic acid were also measured. Each measurement was performed in triplicate, and the results are expressed as mean ± standard error (SE). The percentage inhibition (PI) of the DPPH radical was calculated according to the following formula:

PI = [((*A*C − *A*T)/*A*C) × 100]

where *A*C = absorbance of the control at t = 0 min and *A*T = absorbance of the sample +DPPH at t = 16 min.

### 2.5. In Vitro Antibacterial Activity

The minimum inhibitory concentration (MIC) of DEO was assessed using a resazurin-based turbidimetric microdilution method [27]. A stock solution containing 100 mg/mL of Dill essential oil was prepared using sterile distilled water. The initial column of a 96-well plate received 100 µL of this solution, which was then serially diluted twice across Columns 2 to 10. Following this, 50 µL of the stock bacterial suspension (6 log CFU/mL) was added to each well in Columns 1–10, yielding a final concentration of 5–5.7 log CFU/mL. The tested Gram-negative strains included *Escherichia coli* ATCC 25,922 and four field isolates of Salmonella enterica—BS26, BS29, N7, and N9—that were previously isolated and identified by Sabeq et al. (2022) [28] and Gamil et al. (2024) [29]. The Gram-positive bacterial panel consisted of *Listeria monocytogenes* ATCC 19,115 and two strains of *Staphylococcus aureus*, ST62 and NC15. Column 11 contained 100 µL of the diluted standardized inoculum, while Column 12 held 100 µL of medium broth as a sterility control. After incubation at 37 °C for 24 h, 30 µL of resazurin (0.015%) was introduced to each well. The wells were then incubated for an additional 2 to 4 h to monitor color changes. Columns that retained the blue resazurin color and showed no change at the end of the incubation period were considered to have surpassed the MIC value. To determine the minimum biocidal concentration (MBC), the contents of wells with concentrations exceeding the MIC value were immediately plated.

### 2.6. Fish Sample Collection and Preparation

**Figure 1:**
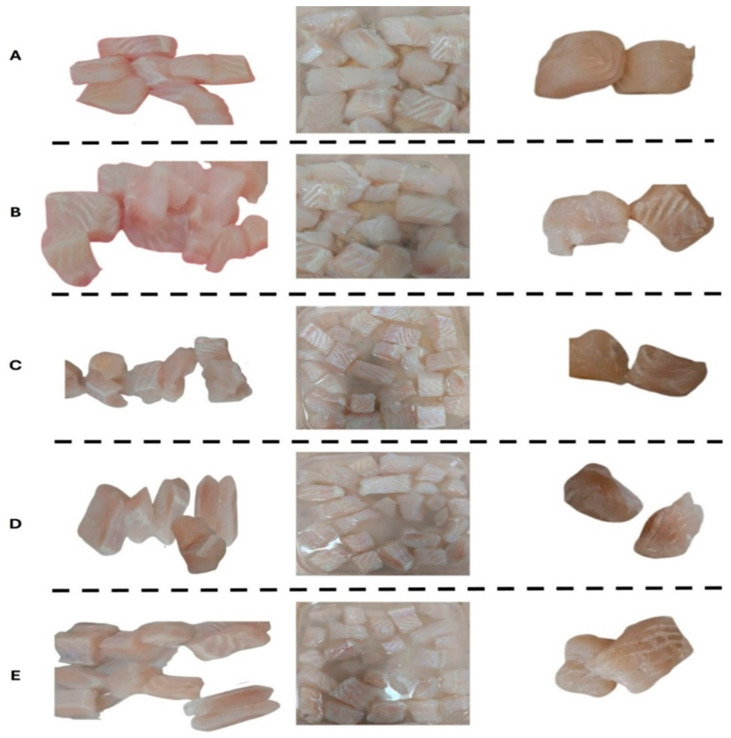
Fish sample preparation and treatment using BHT and three different concentrations of Dill essential oil. (**A**) Control group; (**B**) BH-treated group; (**C**) Dill essential oil-treated group (200 ppm); (**D**) Dill essential oil-treated group (2000 ppm); and (**E**) Dill essential oil-treated group (4000 ppm).

The research utilized fresh Basa fish fillets (*Pangasius bocourti*) acquired from a seafood supplier in Banha City, Egypt, during October and November 2024. A total of 7.5 kg of fillets were promptly transported to the laboratory in ice at 0 °C. The fillets, weighing 300 g each, were randomly allocated into five groups. The control samples underwent immersion in sterile distilled water, while BHT-treated fillets were submerged in a 200 ppm (*w*/*v*) BHT solution. The DEO dipping solution was created by dissolving DEO in a 0.8% (*w*/*v*) tween 80 solution using a homogenizer mixer (Hielscher, Teltow, Germany) at 1500 rpm for 5 min. The remaining three groups were treated with Dill essential oil (EO) at varying concentrations: 200 ppm (DEO-1), 2000 ppm (DEO-2), and 4000 ppm (DEO-3). All samples were immersed for a duration of 30 min (Figure 1).

After extraction from the dipping solutions, the specimens were allowed to drain on a sanitized sieve for 15 min. They were then placed in dual zipper food-grade low-density polyethylene (LDPE) bags and distributed across 6 monitoring locations. The samples were stored in a programmable cooling incubator at 2.5 ± 0.5 °C (BINDER GmbH, Tuttlingen, Germany). At designated intervals (0, 3, 6, 9, 12, and 15 days post-treatment), the samples underwent analysis for physicochemical properties, microbial load, and sensory attributes throughout the 15-day period. The experiment was conducted in duplicate.

### 2.7. Physicochemical Analysis of Fish Fillets

#### 2.7.1. pH Measurement

In summary, the pH of each fish fillet sample was directly assessed using electrodes from a pH meter (Jenway 3510 pH meter, Cole-Parmer, Staffordshire, UK). The device was calibrated at ambient temperature using three distinct pH levels (10, 4, and 7) in combination with a metal temperature probe [30].

#### 2.7.2. WHC Estimation

The filter paper press method (FPPM) was employed to assess the water-holding capacity (WHC) of fish fillets. This technique involved applying a 5 kg weight for 30 s to compress a 0.2–0.5 g sample on Whatman No. 1 filter paper. The WHC of the meat was determined by calculating the percentage of water retained in the sample after deducting the forced loose moisture from its initial weight [31].

#### 2.7.3. Purge Loss (PL) and Cooking Loss (CL) Estimation

For the assessment of purge loss, two cuboidal samples weighing 50 ± 5 g each were designated. The evaluation of purge loss during various storage intervals was conducted by calculating the percentage decrease in fish fillet weight from the initial measurement taken on the first day of refrigeration (at 0, 3, 6-, 9-, 12-, and 15-days following treatment) [32]. Following the estimation of purge loss at each storage interval, the same two samples were utilized to assess cooking loss (CL). Fish fillets, previously weighed and shaped into cuboids (50 ± 5 g), were individually sealed in thin-walled, heat-resistant plastic bags. These were then immersed in a water bath at 80 °C for 10 min. After heating, the samples were cooled to room temperature using tap water, further chilled to 5 °C in an ice bath, dried, and weighed again. The CL was calculated as the percentage difference between the initial raw weight and the final cooked weight [32].

#### 2.7.4. Warner–Bratzler Shear Force (WBSF)

The Warner–Bratzler Shear Force (WBSF) measurement was conducted on cooked fish fillets utilizing the 3343 Universal Test Device Mono column (Instron, Norwood, MA, USA). The samples were cut perpendicular to the muscle fibers’ longitudinal orientation. The WBSF value, expressed in kilogram-force (KGF), was calculated as the mean of six core measurements taken from each fish fillet sample [33].

#### 2.7.5. Instrumental Color Analysis

The chromometer CR-410 (Konica Minolta Sensing INC., Osaka, Japan) was utilized to measure three color parameters: L*, a*, and b* in the samples. The device was set to the L*, a*, b* color space with illuminant D65, using a 2° observer angle and an 8.0 mm aperture size with a closed cone. Prior to taking measurements, the instrument was calibrated using a standardized white tile. Readings were taken across the cut surface of peeled shrimp after allowing it to bloom for 30 min. The obtained color values were then employed to calculate color saturation (Hue angle (*h*˚) = arctg b*/a*) and color intensity (C = (a*^2^ + b*^2^)^0.5^). For each group, an average of six measurements was taken. Increased Chroma levels signify higher saturation of the sample’s primary Hue, while greater Hue angle (or color intensity) values indicate a reduced amount of meat [34], and the total color difference (ΔE), which indicates the amount of color difference between shrimp before and after storage, was calculated as follows [35]:

(ΔE)=L*−L*02+a* −a* 0 2+b*−b*0212

Whiteness indices (WIs) are mathematical formulas that combine measurements of lightness, yellow, and blue into a single value to assess the level of whiteness [36]. The WI was determined according to the following formula reported in [37]:

$$\mathrm{WI}=100-\;\sqrt{{{\left(100-L\right)}^{2}+a}^{2}+b^{2}}$$

And the yellowness index (YI) measures how much a sample’s surface deviates from pure white in terms of its yellow coloration [20]:

$$\mathrm{YI}=142.86*\frac{b}{L}$$

Browning index (BI) $=\frac{100}{0.17}\left(\frac{a*+1.75L*}{5.645L*+a*-0.012b*}-0.31\right)$ [38]

### 2.8. Fish Fillet Microbial Analysis

The aerobic plate count (APC), coliform count, lactic acid bacteria count, and staphylococcal count of Basa fillets generated from compared groups were assessed over 12 days (0, 3, 6, 9, and 12 days) in a binder incubator (BINDER GmbH, Tuttlingen, Germany) at 2.5 ± 0.5 °C.

#### 2.8.1. Determination of Aerobic Plate Count

The aerobic plate count (APC) in Basa fillet samples was assessed in the same manner as for ground beef products [39]. To prepare each sample, a 10% homogenate was created by combining 10 g of the sample with 90 mL of sterile distilled water using a Stomacher 400R (Seward, West Sussex, UK) under aseptic conditions. The resulting homogenates underwent serial tenfold dilutions in sterile distilled water. For each dilution, 1 mL was spread on the surface of two separate sterile plate count agar plates (Condalab, Madrid Spain). Following inoculation and solidification, the plates were incubated at 37 °C for 24 h [40]. Colonies were counted and reported as log colony-forming units per gram of food (CFU/g).

#### 2.8.2. Lactic Acid Bacteria Count (LAB)

To identify lactic acid bacteria (LAB), researchers utilized Man, Rogosa, and Sharpe medium agar (MRS, HiMedia, Kennett Square, PA, USA) with previously prepared tenfold serial dilutions. The samples were applied using the spread plating technique and then placed in a Gas Pak Jar for anaerobic incubation at 30 °C for a duration of 72 h [39]. The number of colonies was measured and expressed as log CFU/g of food.

#### 2.8.3. Determination of Coliform Count

For coliform enumeration, 1 mL of previously prepared tenfold serial dilutions from Basa fillet homogenates was introduced into two separate sterile Petri dishes containing Violet red bile agar (Himedia Laboratories, Maharashtra, India). The dishes were then incubated at 37 °C [41]. The number of colonies was measured and expressed as log CFU/g of food.

#### 2.8.4. Determination of *Staphylococcus* Count

*Staphylococcus* counts in Basa fillets were determined using the surface-plating method on a Baird Parker agar plate (Oxoid, Hampshire, UK), as previously published for milk [42]. A sterile disposable spreader was employed to evenly distribute one milliliter of each previously prepared serial dilution across a Baird Parker agar plate. The inoculated plates were then placed upright in an incubator for a period ranging from approximately 10 min to one hour, allowing sufficient time for the agar to absorb the inoculum. The injections were inverted and incubated at 37 °C for 48 h [43]. The number of colonies was measured and expressed as log colony-forming units (CFUs) per gram of food.

### 2.9. Sensory Evaluation

Sensory evaluation of raw fish fillets was conducted by ten-member panels that underwent necessary training and testing. The training was considered complete when the panelists were comfortable with the evaluation process and individual scores were within one unit of the mean score. The laboratory assessment involved serving representative fish fillet samples on porcelain plates in an open area, without disclosing the treatment type, with three replicates per sample. With the provision of proper natural light, panelists employed a nine-point hedonic scale to evaluate freshness, assigning scores from 1 to 9 for each attribute based on sensory quality criteria. The evaluation encompassed color, odor, appearance, and texture. Overall sensory quality scores were used to categorize fish fillets as very good, good, acceptable, unacceptable, or bad, corresponding to the scale: 9 (like extremely), 8 (like very much), 7 (like moderately), 6 (like slightly), 5 (neither like nor dislike), 4 (dislike slightly), 3 (dislike moderately), 2 (dislike very much), and 1 (dislike extremely). The samples were evaluated in their natural state without additional preparation [44].

### 2.10. Statistical Analysis

SPSS Version 22 (SPSS Inc. Chicago, IL, USA) was used for data analysis. The effects of antimicrobial dipping, storage periods (1, 3, 6, 9, 12, and 15 d), and their interaction on the fish fillet’s physicochemical, microbiological, and antioxidant attributes were examined using general linear models (GLMs), with fillets being treated as a random variable and antimicrobial dipping and chilling time as fixed effects. The means and standard errors of the results were determined. The statistical model employed Tukey’s b multiple comparison test to assess the impact of antimicrobial dipping compared to the control and compare different monitoring point averages within the same group. Significant differences were defined as *p* < 0.05. All experiments were performed in triplicate, and data are presented as mean ± standard error (SE). For each treatment group and storage day, three fish fillet samples (*n* = 3) were used for microbial and physicochemical analyses, including pH, water-holding capacity, cooking and purge losses, shear force, and color measurements. Antioxidant assays (e.g., DPPH) and MIC determinations were conducted using three independent replicates (*n* = 3) for each concentration. For the sensory evaluation, a trained panel of ten assessors (*n* = 10) evaluated the samples at designated intervals.

Principal component analysis (PCA) was employed to assess the relationships among various physicochemical and microbiological parameters of fish meat samples treated with different antioxidants, including DEO at three concentrations, a control (CON), and BHT (a synthetic antioxidant). The analyzed parameters included color change, pH, water-holding capacity (WHC), CL, PL and microbial assessment. Prior to the PCA, all data were standardized to ensure comparability across variables with different measurement scales. The PCA was conducted using R (FactoMineR package, Version 4.5.0), where the covariance matrix was computed, and eigenvalues and eigenvectors were extracted to determine the principal components (PCs). The selection of significant PCs was based on eigenvalues greater than 1 (Kaiser’s criterion) and scree plot analysis. The contribution of each variable to the total variance was assessed through loading scores, and a biplot was generated to visualize the clustering of treatments and the influence of different parameters. Bartlett’s test of sphericity and the Kaiser–Meyer–Olkin (KMO) measure of sampling adequacy were performed to validate the appropriateness of the PCA, with a KMO value greater than 0.7 indicating suitability. The results of the PCA provided insights into the major factors influencing the quality attributes of fish meat under different antioxidant treatments.

## 3. Results

### 3.1. Dill Essential Oil Chemical Composition

Gas chromatography–mass spectrometry (GC/MS) analysis of DEO provided a comprehensive chemical profile of its constituents, offering insights into its potential functional and therapeutic applications (Table 1). GC/MS analysis revealed the presence of multiple bioactive compounds in DEO, each characterized by distinct retention times and mass spectral patterns. The chemical profile indicated that monoterpenes dominated the volatile fraction and accounted for most of the identified compounds.

### 3.2. Dill Oil Antioxidant Activity

**Figure 2:**
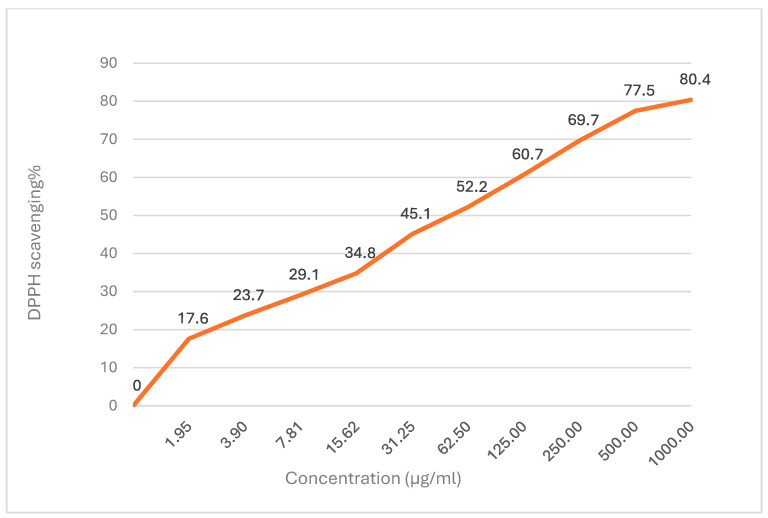
DDPH scavenging % of Dill essential oil.

The antioxidant activity of DEO was evaluated using the DPPH (2,2-diphenyl-1-picrylhydrazyl) radical scavenging assay (Figure 2). The DEO demonstrated dose-dependent antioxidant activity, with DPPH scavenging percentages ranging from 17.6% at 1.95 µg/mL to 80.4% at 1000 µg/mL. The IC50 value, which represents the concentration required to inhibit 50% of DPPH radicals, was determined to be 48.3 ± 0.9 µg/mL.

### 3.3. In Vitro Antimicrobial Assay

**Figure 3:**
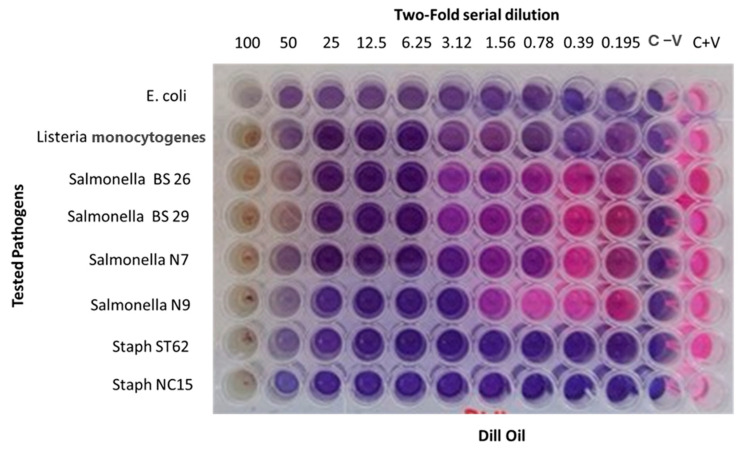
Microdilution assay for determining the minimum inhibitory concentration (MIC) of Dill essential oil (DEO) against bacterial strains.

Dill (*Anethum graveolens* L.) essential oil exhibited moderate antibacterial activity, particularly against Gram-positive bacteria such as *Listeria monocytogenes* and *Staphylococcus aureus*, with MIC values as low as 0.195 mg/mL (Figure 3). The antibacterial activities of DEO on Gram-negative bacteria, including *E. coli* and Salmonella isolates, were less pronounced, requiring higher concentrations (up to 6.25 mg/mL) for growth inhibition. The MIC results revealed varied antimicrobial activity of DEO across the tested bacterial strains. For *Escherichia coli* ATCC 25922, growth inhibition was observed at concentrations ≥ 3.9 mg/mL. For the *Salmonella enterica* isolates BS26 and BS29, the MIC was 6.25 mg/mL, while the MIC of both the N7 and N9 isolates was 3.12 mg/mL. In contrast, the *Listeria monocytogenes* ATCC 19,115 and *Staphylococcus aureus* field isolates ST62 and NC15 were more sensitive, with MICs as low as 0.195 mg/mL. These concentrations were calculated based on a stock solution of DEO prepared at 100 mg/mL (Figure 3).

### 3.4. Physicochemical Analysis of Fish Fillets

**Figure 4:**
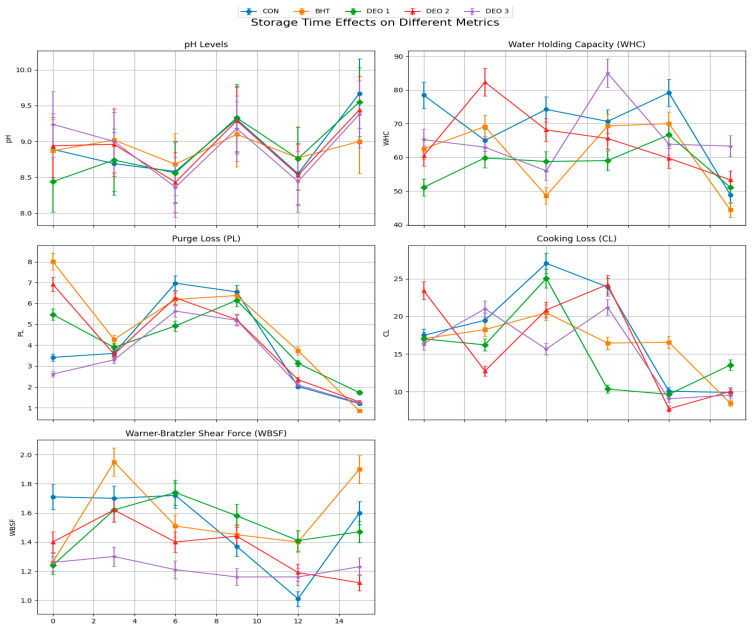
Changes in physicochemical parameters over time in fish subjected to different Dill essential oil treatments.

Statistical results indicated a significant interaction between treatment and storage time, affecting all estimated physicochemical attributes and color (*p* < 0.05). DEO treatment influenced all physicochemical attributes except CL and DL. Additionally, all physicochemical attributes were significantly affected by the storage interval (*p* < 0.05). The pH values of the fish samples varied significantly across the different treatment groups and storage times (*p* < 0.001) (Figure 4). On Day Zero, the highest pH was observed in the DEO-3 group (9.24), followed by DEO-2 (8.94) and BHT (8.87), whereas the lowest pH was recorded in the DEO-1 group (8.44). By the third day, the pH values remained significantly different, with BHT-treated samples exhibiting the highest pH (9.02) and DEO-1 samples displaying a lower pH (8.74) (*p* < 0.05). On the sixth day post-treatment, All DEO-supplied groups, particularly DEO-3, had lower pH values than both the control and BHT (*p* < 0.05). By the ninth day, the control and DEO-1 groups had the highest pH values (9.31 and 9.33, respectively), while BHT-treated samples exhibited a significantly lower pH (9.10) (*p* < 0.05), indicating better preservation. On the twelfth day, all groups exhibited comparable pH values, ranging from 8.44 to 8.77, demonstrating a general stabilization of pH across treatments. However, on day 15, the control group exhibited the highest pH (9.67), indicative of spoilage progression, whereas the BHT-treated samples maintained a significantly lower pH (9.00), reinforcing their effectiveness in preserving fish quality. The overall trends suggest that the BHT and DEO treatments influenced pH fluctuations over time, with BHT being more effective in maintaining stability, whereas DEO treatments showed concentration-dependent variations. Moreover, WHC values varied on the first day, while all other groups were not different from the control (*p* > 0.05). On the third day post-treatment, DEO-2-supplied fish exhibited the highest WHC value compared to all other groups (*p* < 0.05). On all of the days following post-treatment, the BHT- and DEO-treated groups had similar WHC values compared to the control (*p* > 0.05). The longer storage period decreased the WHC values in the control fish, which was obvious in the last storage period compared to the first day (*p* < 0.05). Compared to the first day, all other fish treated with BHT or different DEO levels, particularly DEO-1, exhibited similar WHC values across the entire storage period (*p* > 0.05). Purge loss was not affected by BHT and/or DEO treatment (*p* > 0.05), except on the third and ninth days post-treatment where DEO-3- and BHT-supplied fish exhibited higher purge loss than the control (*p* < 0.05), respectively. All DEO-treated fish exhibited similar purge loss (PL) values across the entire storage period compared to the first day (*p* > 0.05). However, the control fish showed an ascending curve that reached the peak at six days post-treatment (*p* < 0.05) and then dropped to similar loss values compared to the first day (*p* > 0.05). BHT-supplied fish, compared to the first day, exhibited a declining PL curve and reached the lowest losses at 12 and 15 days post-treatment (*p* < 0.05). Cooking losses (CLs) were not affected by BHT and/or DEO treatment (*p* > 0.05), except on the 12th day post-treatment where BHT-supplied fish exhibited higher loss than all other groups (*p* < 0.05). Compared to the first day, all groups did not show significant differences in CLs across the entire storage period (*p* > 0.05), except DEO-1- and DEO-2-supplied fish (*p* < 0.05). The DEO-1-supplied fish showed an ascending CL curve and reached the peak a day six post-treatment, followed by a sharp declining trend compared to the first day (*p* < 0.05). While DEO-2-supplied fish had similar CL values until the ninth day post-treatment (*p* > 0.05), followed by a sharp lower CL trend on the last two storage periods compared to the first day (*p* < 0.05).

The Warner–Bratzler Shear Force (WBSF) values were assessed at multiple time points to evaluate the impact of different antioxidant treatments (control, BHT, DEO-1, DEO-2, and DEO-3) on texture retention (Figure 4). In the initial measurement (D-Zero), the control group exhibited the highest WBSF value (1.71), indicating a firmer texture. In contrast, fish treated with BHT (1.26), DEO-1 (1.24), and DEO-3 (1.26) demonstrated significantly lower WBSF values, suggesting an initial softening effect compared with the control (*p* < 0.05). Over time, the control group exhibited a declining WBSF trend, reaching its lowest value at D-12 (1.01) before slightly increasing at D-15 (1.60). This pattern indicates loss of firmness, likely due to protein degradation and structural changes during storage. The BHT-treated group showed fluctuations in WBSF, with a notable peak at D-3 (1.95) and another increase at D-15 (1.90). This suggests that BHT contributed to the preservation of structural integrity, delaying the softening process. DEO-1-treated samples exhibited moderate fluctuations, with a peak at D-9 (1.58) and a subsequent increase at D-15 (1.47), indicating partial resistance to texture deterioration. Similarly, DEO-2-treated fish maintained a relatively stable pattern, peaking at D-12 (1.41) and decreasing at D-15 (1.12), suggesting that DEO-2 delayed texture degradation until later storage periods. The most distinct pattern was observed in the DEO-3-treated group, which demonstrated an ascending WBSF trend, reaching its peak at D-6 (1.74) before declining at D-9 (1.16). This suggests that DEO-3 initially enhanced muscle firmness, but this effect diminished after prolonged storage. Statistical analysis confirmed that treatment (G), time (T), and their interaction (G*T) significantly influenced the WBSF values (*p* < 0.001), highlighting the role of antioxidant supplementation in texture modification.

**Figure 5:**
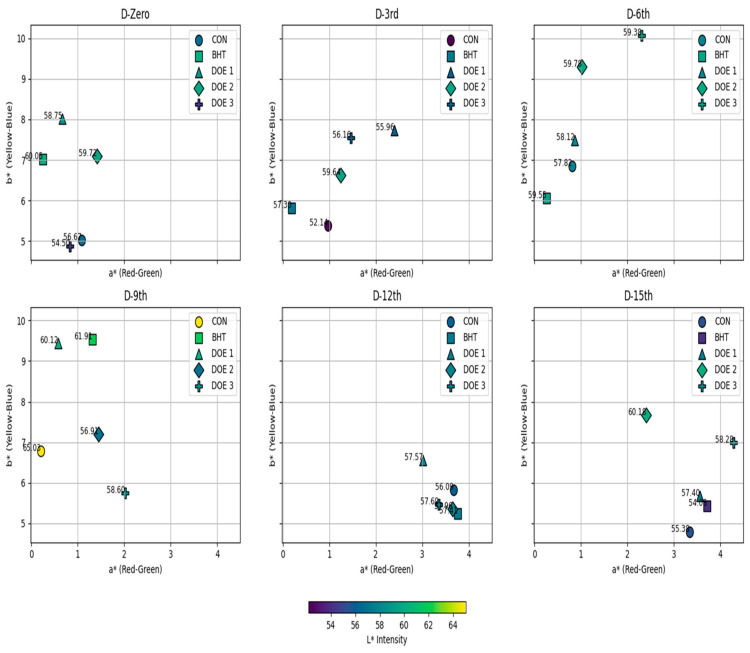
Changes in color parameters of fish meat samples subjected to different treatments over a 15-day storage period. a* (Red–Green Axis): measures the degree of redness (+a*) or greenness (−a*), which is primarily influenced by myoglobin oxidation; b* (Yellow–Blue Axis): represents yellowness (+b*) or blueness (−b*), which is often associated with lipid oxidation and pigment degradation; L* (Lightness Intensity): indicated by the color gradient, with darker shades representing lower L* values and lighter shades indicating higher L* values.

Concerning meat color, it was noted that DEO-3-supplied fish exhibited the lowest lightness (L*) (*p* < 0.05) on the first day; at the same time, both the BHT group and other DEO-supplied groups had higher L* compared to the control (*p* < 0.05) (Figure 5). On the third day post-treatment, the BHT group and all DEO-supplied groups had higher L* compared to the control, particularly DEO-2 (*p* < 0.05). On the ninth day post-treatment, it was noted that DEO-2-supplied fish exhibited the lowest L* (*p* < 0.05), while all other groups were not different from the control (*p* > 0.05). On the twelfth day post-treatment, all groups had higher L* (*p* < 0.05) compared to the control. On the 15th day post-treatment, it was noted that L* sharply decreased in BHT-supplied fish (*p* < 0.05), while DEO-supplied fish displayed higher values compared to the control, clearly in DEO-2 (*p* < 0.05). Compared to the first day, all fish groups, except DEO-2-supplied fish, changed with a longer storage period (*p* < 0.05). Also, all fish groups displayed lower L* values on the third day post-treatment (*p* < 0.05) compared to the first day, and then started to ascend followed by declining curves that reached their peaks either on the sixth day post-treatment in DEO-3 or on the ninth day post-treatment in the control, BHT, and DEO-1-supplied fish (*p* < 0.05). Moreover, the redness index (a*) was not affected by any of the treatments on the first day compared to the control (*p* > 0.05) (the redness index was higher in DEO-2 than the BHT-supplied fish). On the following days, changes started to appear where the BHT-, and DEO-1-treated fish had lower and higher a* values compared to the control, respectively (*p* < 0.05). On the sixth day post-treatment, the BHT-treated fish still had a lower a* value, while the DEO-3-supplied fish exhibited the highest a* value compared to the control (*p* < 0.05). On the ninth day post-treatment, all treated fish groups, particularly DEO-3, exhibited higher a* values compared to the control group (*p* < 0.05) with a longer storage period; the a* value increase in the control fish was obvious on the last two storage periods compared to the first day (*p* < 0.05). Both the BHT group and all of the DEO-supplied groups generated an ascending curve that reached the highest values at either day 12 or 15 post-treatment, particularly BHT and DEO-1 compared to the first day (*p* < 0.05) (Figure 5). Both the BHT- and DEO-supplied fish, except DEO-3, showed higher yellowness (b*) values compared to the control on the first day (*p* < 0.05). On the third day post-treatment, both DEO-1 and DEO-3 exhibited the highest b* values compared to the control and other fish-treated groups (*p* < 0.05). On day six post-treatment, both DEO-2 and DEO-3 exhibited the highest b* values compared to the control and other fish-treated groups (*p* < 0.05). On the ninth day post-treatment, while both the BHT- and DEO-1-supplied groups showed higher b* values compared to the control, DEO-3 exhibited the lowest b* value (*p* < 0.05). On the twelfth day post-treatment, all fish-treated groups exhibited a sharp decrease in b* value compared to the control, except the DEO-1-supplied fish, which showed the highest b* value compared to the control (*p* < 0.05). On the 15th day post-treatment, it was noted that the b* value increased in all fish-treated groups compared to the control (*p* < 0.05). With a longer storage period, the control fish showed an ascending curve that reached the peak at the middle storage periods, six and nine days post-treatment (*p* < 0.05), and then dropped to similar b* values of the first day (*p* > 0.05). Across the entire storage period, except the ninth day, the BHT-supplied fish exhibited declining b* values compared to the first day (*p* < 0.05). Compared to the first day, the DEO-1-supplied group showed stable yellowness values that dropped at the last storage period, 15 days post-treatment (*p* < 0.05). Across the storage period, DEO-2 exhibited comparable b* values to the first day, but higher and lower values were recorded on the sixth and twelfth days post-treatment (*p* < 0.05). With longer storage, the DEO-3-supplied fish displayed an increasing b* trend compared to the first day, which was apparent on the third, sixth, and fifteenth days post-treatment (*p* < 0.05) (Figure 5).

**Figure 6:**
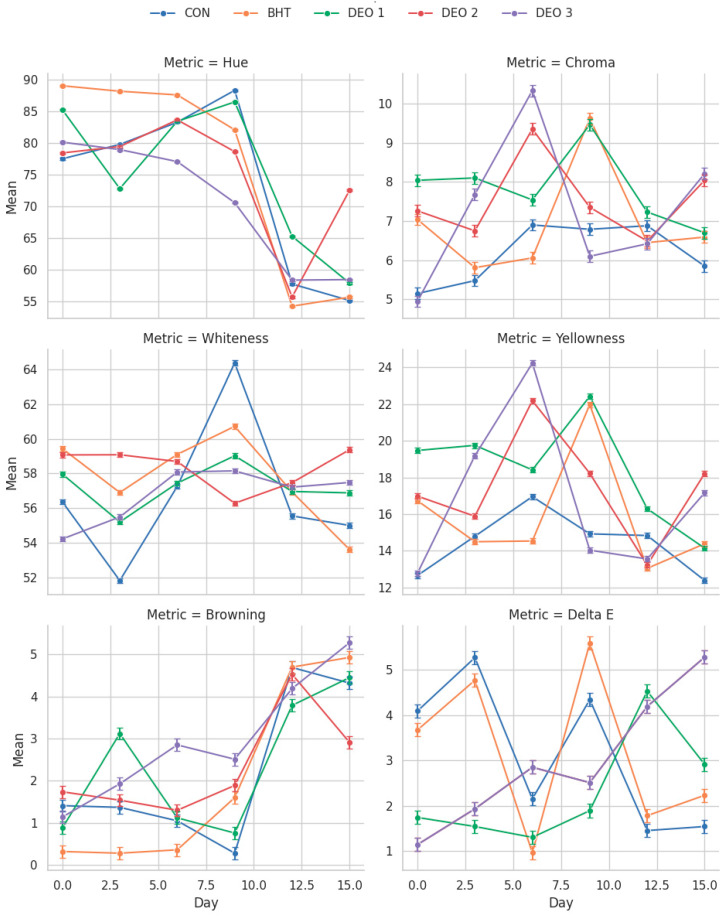
Changes in color stability parameters of fish meat during storage under different Dill essential oil treatments.

Hue (color saturation): It was noted that the BHT-treated fish exhibited a higher Hue than the control and DEO-treated fish from the first day up to the sixth day post-treatment (*p* < 0.05). On the third and sixth days post-treatment, it was noted that the DEO-1- and DEO-3-supplied fish had the lowest Hue than all the other groups, respectively (*p* < 0.05). On the ninth day post-treatment, it was noted that all fish-treated groups had a lower Hue value than the control, particularly the DEO-3-supplied fish (*p* < 0.05). On the twelfth and fifteenth days post-treatment, the DEO-1-supplied and all DEO-treated fish showed a higher Hue, respectively (*p* < 0.05), compared to the control and BHT-treated fish groups. With a longer storage period, the control fish showed an ascending curve that reached the peak on the ninth day post-treatment (*p* < 0.05), and then the curve sharply decreased until the completion of the storage period (*p* < 0.05) compared to the first day. Both the BHT- and DEO-3-supplied fish, compared to the first day, exhibited a declining Hue curve and reached the lowest losses at 12 and 15 days post-treatment (*p* < 0.05). Compared to the first day, the DEO-2-supplied fish had a comparable Hue during the entire storage period (*p* > 0.05), except the lowest value was observed at 12 days post-treatment (*p* < 0.05) (Figure 6).

Chroma: Compared to the control group on the first day, both the BHT- and DEO-supplied fish groups, except DEO-3, exhibited a higher Chroma (*p* < 0.05). This higher Chroma trend was also noticed in the DEO-1- and DEO-3-supplied groups on the third day post-treatment compared to the control, particularly DEO-1. Six days post-treatment, the DEO-2- and DEO-3-supplied groups exhibited a higher Chroma value (*p* < 0.05) than the other groups. On the ninth day post-treatment, both the BHT- and DEO-1-supplied groups exhibited a higher Chroma value (*p* < 0.05) than all other groups. On the twelfth day, all groups differed, where DEO-1-supplied fish had higher values while other treated groups had lower Chroma values compared to the control (*p* < 0.05). Similarly, on the 15th day post-treatment, the BHT- and DEO-1-supplied groups exhibited a lower Chroma value, but the DEO-2- and DEO-3-supplied groups exhibited a higher Chroma value than the control (*p* < 0.05). With a longer storage period, it was noted that both the control and DEO-1-supplied groups did not show differences in Chroma across the entire storage period (*p* > 0.05), while the BHT group exhibited a sharp declining Chroma curve on the third and sixth days post-treatment (*p* < 0.05). Compared to the first day, DEO-2 exhibited a constant curve along the storage experiment (*p* > 0.05) but had a peak on the sixth day post-treatment (*p* < 0.05). While DEO-3-supplied fish showed an ascending curve until six days post-treatment (*p* < 0.05), and then a declining curve compared to the first day (*p* < 0.05) (Figure 6).

Regarding the whiteness index, on the first day, the BHT- and DEO-2 treated groups had significantly higher WI values than the other groups (*p* < 0.05). The DEO-2-fortified group exhibited substantially greater WI values than the other groups on the third and fifteenth days of storage (*p* < 0.05). However, the WI values of the DEO-2-treated group decreased significantly on the ninth day of storage in comparison to the control group (*p* < 0.05). Furthermore, all treated groups, regardless of BHT or DEO, exhibited significantly elevated WI values on the 12th day of cold storage in comparison to the control group (*p* < 0.05). It was observed that the WI values across groups, except the DEO-2-fortified group, displayed a comparable trend. On the first day, there was a downward trend in WI values and then an upward trend, reaching the highest values on days 6 and 9 in the DEO-3-fortified groups and day 9 only in the remaining groups. Then, the WI value decreases until the end of the storage period (Figure 6). For the yellowness index, the yellowness index (YI) in both the BHT- and DEO-1-fortified groups demonstrated a descending trend from the third day, reaching its maximum values on the ninth day and subsequently decreasing until the end of the storage period. Simultaneously, the DEO-2- and DEO-3-fortified groups exhibited a comparable trend as previously observed and achieved their peak YI values on the sixth and fifteenth days of storage. In comparison to other groups, on the sixth day of storage, both the DEO-2- and DEO-3-fortified groups showed the highest YI values (*p* < 0.05). Moreover, the BHT- and DEO-1-fortified groups had higher YI values than the other groups on the 9th day of storage (*p* < 0.05), but DEO-1 displayed a substantially greater YI value than the other groups on the 12th day of cold storage. Furthermore, for the browning index, on the first day of cold storage, the browning index (BI) for the DEO-2-fortified group exhibited a statistically significant increase (*p* < 0.05) in comparison to the BHT-fortified group. The DEO-1-fortified group exhibited the highest BI value, whereas the BHT-fortified group displayed the lowest BI value in comparison to the other groups (*p* < 0.05) on the third day of cold storage. Furthermore, the DEO-3-fortified group exhibited the highest BI value relative to the other groups (*p* < 0.05) on the 6th, 9th, and 15th days of cold storage; however, on day 12, both the control and BHT-treated groups demonstrated the highest BI values compared to the other groups (*p* < 0.05). The browning index values within the groups exhibited a similar pattern, with a decrease during cold storage and an upward trend until they reached their maximum level on day 12 for the control, BHT-fortified group, and DEO-2-fortified group, and on day 15 for both the DEO-1- and DEO-3-fortified groups (Figure 6). The Delta E (∆E) level of the DEO-3-treated group was lower than that of the BHT-treated group on the initial day of cold storage (*p* < 0.05). Nevertheless, the ∆E level of both the BHT- and DEO-1-treated groups increased in comparison to the other treated groups on the third day of cold storage (*p* < 0.05) (Figure 6). Furthermore, the DEO-3-treated groups exhibited the maximum ∆E level in comparison to the other treated groups (*p* < 0.05) on days 6 and 15 of cold storage. On the ninth day of cold storage, the ∆E levels of both the BHT- and DEO-1-treated groups were significantly higher than those of the other treated groups (*p* < 0.05). On the twelfth day of cold storage, DEO-2 exhibited higher ∆E levels than those of the other groups (*p* < 0.05). The ∆E levels within the group exhibited a similar pattern to that of WI, YI, and BI, as they initially decreased and then increased in an ascending manner until they reached their maximum levels on day 9 for the BHT- and DEO-1-treated groups, day 12 for the DEO-2-treated groups, and day 15 for the DEO-3-treated groups, respectively.

### 3.5. Fish Fillet Microbial Analysis

**Figure 7:**
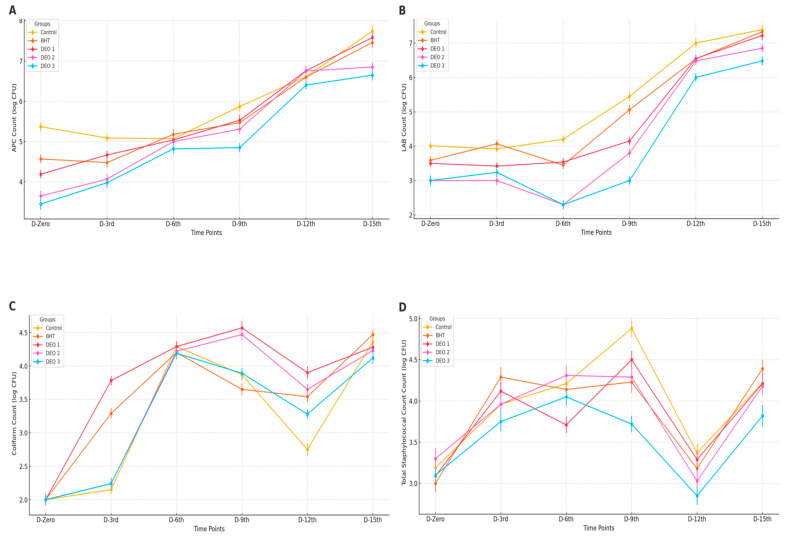
Microbial analysis of fish fillets stored at refrigeration (4 °C) for 15 days after treatment with Dill essential oil (DEO) at three concentrations (DEO-1, DEO-2, and DEO-3) compared with the control and BHT groups. (**A**) Aerobic plate count (APC); (**B**) lactic acid bacteria (LAB) count; (**C**) coliform count; and (**D**) total staphylococcal count. Error Bars represent the standard error (SE) of the mean microbial counts (log CFU/g) at each storage period.

The application of BHT and/or DEO generally did not yield significant effects on APC (*p* > 0.05) from the first day to day 15 post-treatment, with one notable exception. On day nine after treatment, higher DEO concentrations were found to reduce APC below 5 log CFU/g (*p* < 0.05) compared with the control and other treated fish groups. Although not statistically significant, the DEO-treated groups, particularly DEO-3, exhibited numerically lower APC by approximately one log CFU/g compared to the control group (*p* > 0.05) until the ninth day post-treatment (*p* < 0.05). As the storage duration increased, all groups, including the positive control and BHT-treated samples, showed an increasing trend, reaching over 6 log CFU/g at 12 and 15 d post-treatment (*p* < 0.05) (Figure 7A). Likewise, the BHT and/or DEO treatments did not significantly affect LAB (*p* > 0.05) during the initial 15 d post-treatment. However, with increased storage time, all groups, including the positive and negative controls, displayed an upward trend, exceeding 6 log CFU/g at 12- and 15- days post-treatment (*p* < 0.05). Although the DEO-treated groups, especially DEO-3, showed numerically lower lactic acid bacteria counts (~1.5 log CFU/g) on day 9 compared to the control, these reductions were not statistically significant (*p* > 0.05). Therefore, no definitive antimicrobial effect can be concluded at this storage point. Nevertheless, by the twelfth day post-treatment, LAB counts in all groups, including DEO-treated fish, surpassed six logs CFU/g (*p* < 0.05) (Figure 7B). The BHT and/or DEO treatments generally did not affect coliform levels (*p* > 0.05), with two exceptions: on the third and ninth days after treatment, fish treated with DEO-2 and BHT exhibited higher coliform counts than the control and other treated groups (*p* < 0.05). As the storage time increased, an upward trend in coliform counts was observed across all fish groups, particularly in the negative control (*p* < 0.05) (Figure 7C). Regarding the impact on staphylococcal counts, all groups showed similar counts, a pattern that was repeated in the final two post-treatment storage periods. However, variations emerged during the intermediate periods. On the third day post-treatment, the BHT-treated fish displayed higher staphylococcal counts than those of the control. By the sixth day, DEO-1 showed differences, and on the ninth day, all DEO-treated groups exhibited lower counts than the control and other groups (*p* < 0.05). As storage time increased, most groups maintained stable staphylococcal counts relative to day one, except for the control and BHT-treated fish. These two groups showed significant increases (*p* < 0.05) on the 9th and 15th days post-treatment. Notably, the DEO-treated fish remained unaffected by extended storage (*p* > 0.05) (Figure 7D).

### 3.6. Sensory Evaluation

**Figure 8:**
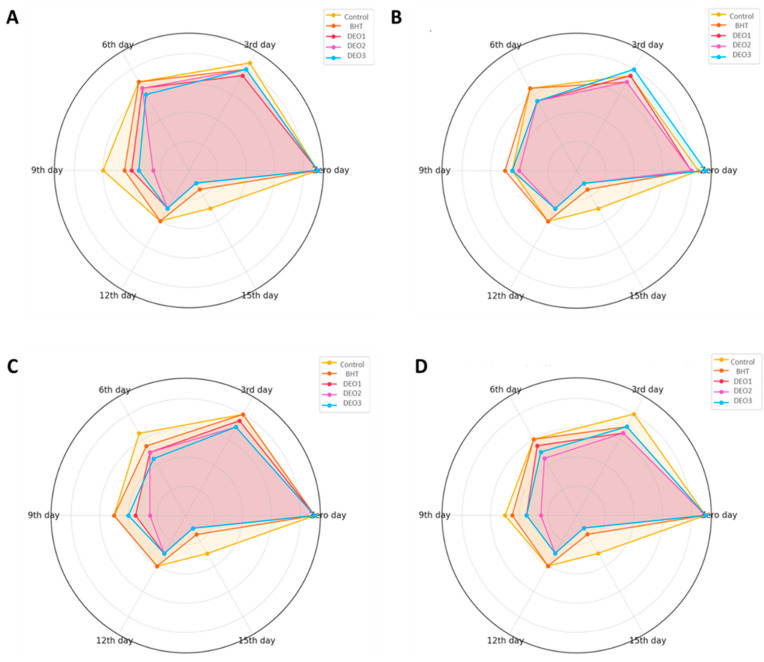
Sensory evaluation of fish meat treated with different concentrations of Dill essential oil compared to BHT and the control group over a 15-day storage period. The sensory attributes assessed included odor (**A**), color (**B**), texture (**C**), and overall acceptability (**D**) at different time intervals.

Figure 8 illustrates a detailed comparison of the sensory evaluation results for fish meat subjected to various treatments. These treatments included three different concentrations of DEO, butylated hydroxytoluene (BHT), and a control. The assessment, conducted over a storage period, examined four key sensory attributes: odor, color, texture, and overall acceptability. Evaluations were performed at the start of the experiment (Day Zero) and subsequently on days 3, 6, 9, 12, and 15. As anticipated, all treatment groups exhibited a gradual decrease in sensory scores throughout the storage period. This decline was attributed to the natural degradation of fish meat quality, primarily due to processes such as lipid oxidation and microbial growth. In the odor assessment, all the treatment groups initially showed comparable scores. However, the control group experienced the quickest deterioration in odor quality, especially after day 6. While the BHT-treated samples maintained better odor scores than the control, they still showed a consistent decline by day 15. The DEO treatments demonstrated a concentration-dependent effect, with DEO-3 preserving the odor quality for the longest period, followed by DEO-2 and DEO-1. The coloration changed over time. By the ninth day, both the control and BHT groups exhibited a significant decrease in color scores. In contrast, the samples treated with Dill, particularly DEO-3, demonstrated superior color retention. Texture analysis revealed a gradual decrease in firmness and overall textural quality across all treatments over time. The samples in the control group softened most rapidly, whereas those treated with DEO-3 maintained a firmer consistency for an extended period. A comprehensive sensory evaluation, which integrates individual sensory characteristics into a single assessment, was initially favorable for all samples. However, by day 9, the acceptability ratings of the control group had decreased significantly. Although the BHT-treated samples outperformed the control, they showed a gradual decline. In contrast, the Dill-treated groups demonstrated superior preservation of the overall sensory quality, with DEO-3 consistently receiving the highest scores throughout the experiment.

### 3.7. Correlations Between Physicochemical Parameters

**Figure 9:**
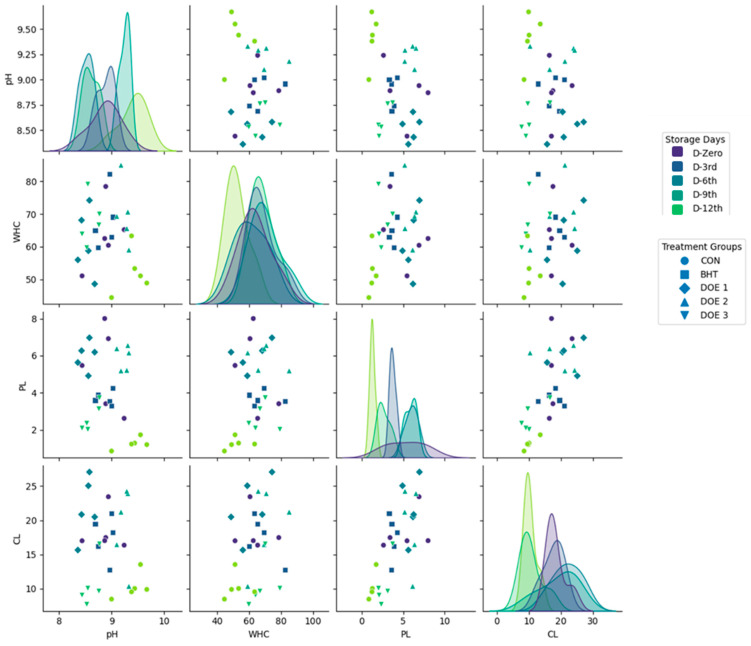
Scatter plot matrix of physicochemical parameters (pH, WHC, PL, and CL) in stored fish meat under different treatment groups and storage durations. Each subplot represents the relationship between two variables, with diagonal plots displaying kernel density estimations (KDEs) for distribution analysis. Storage days are color-coded (D-Zero, D-3rd, D-6th, D-9th, and D-12th), while treatment groups are represented by different marker shapes (● CON, ■ BHT, ♦ DEO-1, ▲ DEO-2, and ▼ DEO-3).

The scatter plot matrix reveals several key relationships among the physicochemical parameters analyzed (Figure 9). The correlation between pH and WHC appears weak or inconsistent. Additionally, a moderate negative correlation was observed between WHC and CL, whereby samples with higher WHC tend to exhibit lower CLs. Furthermore, a positive correlation between PL and CL underscores the interconnected nature of protein integrity and CL.

### 3.8. PCA Analysis

**Figure 10:**
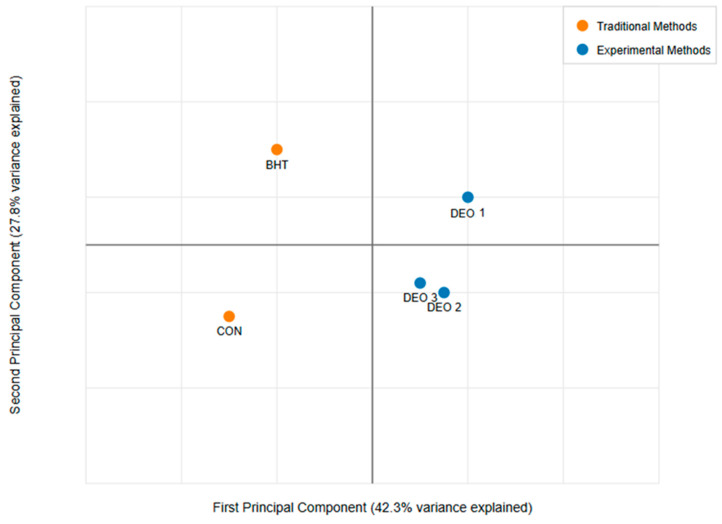
PCA analysis of treatment effects.

The principal component analysis (PCA) presented in Figure 10 illustrates the distribution of different treatment groups—control (CON), BHT (a synthetic antioxidant), and three concentrations of Dill essential oil (DEO-1, DEO-2, and DEO-3)—based on key physicochemical and microbiological parameters, including color change, pH, water-holding capacity (WHC), CL, PL, and microbial load. The first principal component (PC1) accounts for 42.3% of the total variance, while the second principal component (PC2) explains 27.8%, collectively capturing a substantial portion of the dataset’s variability. The traditional treatments, CON and BHT, are positioned in distinct quadrants. The CON group, located in the lower-left quadrant, suggests minimal alterations in physicochemical properties, likely reflecting untreated fish meat’s natural deterioration over time. In contrast, BHT appears in the upper-left quadrant, indicating a different trajectory in response to antioxidant treatment, possibly due to its effects on oxidative stability and microbiological control. The experimental treatments (DEO-1, DEO-2, and DEO-3) cluster within the right half of the PCA plot, signifying distinct physicochemical and microbiological characteristics compared to traditional treatments. DEO-1, located in the upper-right quadrant, demonstrates a unique response, potentially reflecting stronger antioxidant or antimicrobial effects at this concentration. DEO-2 and DEO-3, closely positioned in the lower-right quadrant, indicate similar influences on fish meat properties, suggesting a trend in treatment response as the DEO concentration increases.

## 4. Discussion

The current study aimed to assess the preservation effects of *Anethum graveolens* essential oil (DEO) on the quality and shelf life of Basa fish fillets in refrigerated storage. The main components of Dill essential oil are monoterpenes, primarily α-phellandrene, d-limonene, carvone, and Dill ether. These findings align with those of [45], who found that carvone (42.47%), limonene (29.04%), and α-phellandrene (13.12%) are essential components of Dill essential oil. Similarly, ref. [46] identified significant concentrations of carvone (34.33%), α-phellandrene (22.03%), Dill ether (18.84%), limonene (6.93%), and Dill apiol (5.01%) as primary constituents. In addition, refs. [8,47] also found that the most common monoterpenes in DEO are carvone and limonene. Nevertheless, ref. [48] disclosed that the majority of the components of DEO are dillapiole (44.01%), d-limonene (19.47%), and carvotanacetone (14.03%). The variations in the concentration and composition of various components can be ascribed to varied weather conditions and geographical regions, as well as metabolism, maturity, and the portion of the plant from which the essential oils (EOs) are extracted [49]. Furthermore, harvesting time, storage conditions, and extraction techniques all have an impact on essential oil yield and composition [50,51]. Although DEO lacks classical phenolic compounds, its moderate DPPH scavenging activity may be attributed to oxygenated monoterpenes such as carvone and limonene, which have demonstrated radical scavenging activity in other studies despite not possessing aromatic hydroxyl groups [52,53]. So, DEO acts as a promising source of antioxidants due to its high level of phenolic compounds. The DPPH antioxidant assay results indicated a concentration-dependent variation in the suppression of DPPH radicals by the DEO. The antioxidant activity may be ascribed to the presence of monoterpenes in DEO, which may act as radical scavenging agents. The majority of earlier experiments concluded that the essential oils contained monoterpene hydrocarbons, oxygenated monoterpenes, and/or sesquiterpenes, which have strong antioxidative properties [53]. Limonene from the class of monoterpene phenols has been reported to possess antioxidant activity [54]. Thus, the current high content of limonene component might contribute to estimated high antioxidant activity. Limonene is listed in the Code of Federal Regulations as generally recognized as a safe (GRAS) antioxidant in human foodstuff [55].

The DEO demonstrated a lower antimicrobial potential on Gram-negative bacteria, particularly *Salmonella* spp., in the resazurin-based turbidimetric assay. Nevertheless, it demonstrated a slightly higher antimicrobial potential on *E. coli* at 0.098 mg/mL than the value reported by Mujović et al. (2024) [8], who demonstrated that the MIC of DEO for *E. coli* was 28.41 µL/mL. Conversely, Gram-positive bacteria demonstrated a higher degree of susceptibility to DEO than Gram-negative bacteria, which is likely attributable to variations in cell wall structure. DEO flavonoids can form a complex with bacteria’s outer membrane and soluble proteins that are related to it [56]. Nevertheless, the synergistic effect of beneficial essential oil components has previously been shown to be effective in terms of antibacterial activity [57]. Gram-negative bacteria have both an outer membrane and a periplasmic space, whereas gram-positive bacteria lack either of these components. This membrane stops hydrophiles from entering the bacteria. The periplasmic region includes large enzymes capable of decomposing foreign substances [56,58]. These findings align with previous studies highlighting dill oil’s moderate antimicrobial efficacy against foodborne pathogens [8,59]. Biological activities of EOs are related to their chemical compositions [60]. The current principal constituents of DEO are monoterpenes, notably limonene and carvone, which have potent antibacterial effects [8,61,62]. Monoterpenes lipophilic characteristics can permeate cell membranes, enhance fluidity, and inhibit embedded enzymes [63]. Current DEO promising antimicrobial activities against multidrug resistant pathogens support the potential application as a natural antimicrobial agent in food preservation and safety strategies. 

Fish muscle is unlike mammalian muscle, which experiences a significant post-mortem pH decline due to glycogen breakdown into lactic acid and often exhibits a more moderate pH drop due to its lower glycogen reserve [64]. The higher initial pH of DEO-3-treated fish may be attributed to the buffering capacity of certain bioactive compounds in DEO, such as terpenes and phenolic compounds, which can neutralize the acidic metabolites produced during post-mortem changes [8]. Another justification for higher pH might be attributed to the antimicrobial activity of DEO. By inhibiting the growth of native microflora, including current estimated *LAB*, which is capable of acidic byproduct production, DEO treatment could result in a relative increase in pH compared with untreated samples. This DEO antimicrobial effect has also been documented in previous studies on fungal pathogens, where DEO disrupted cellular processes and inhibited acid production [65]. BHT-treated samples showed the highest pH, especially from the 3rd to 6th days compared to the control and DEO-treated groups. This may be due to the antioxidant properties of synthetic BHT, which likely prevents the formation of acidic byproducts from lipid oxidation and proteolysis, maintaining a higher pH [66]. DEO contains bioactive compounds with similar antioxidant properties, but these natural compounds may degrade over time, leading to a decrease in antioxidative and antimicrobial efficacy. The degradation of natural compounds might trigger spoilage involving proteolytic activity and lipid oxidation, resulting in acidic metabolites with a decreased pH. Previous research indicated that while DEO exhibits initial antimicrobial and antioxidant effects, its efficacy diminished with extended storage, leading to quality degradation of fish products [8]. In the untreated control samples and those treated with low concentrations of DEO (DEO-1), the higher pH observed during later storage stages (days 9–15) is likely due to microbial spoilage. Microbial activity leads to the production of basic compounds such as ammonia and biogenic amines, resulting in increased pH. Studies have shown that inadequate antioxidant protection allows microbial proliferation and spoilage, contributing to pH elevation in stored fish products [67]. Higher concentrations of DEO (DEO-3) appeared to be more effective in inhibiting oxidative and proteolytic processes, maintaining muscle quality, and preventing pH fluctuations. The enhanced preservation effect at higher DEO concentrations is supported by studies demonstrating that increased levels of natural extracts can improve the shelf life and quality of fish products during storage [68]. The current study indicates that DEO may enhance the water-holding capacity of fish muscle. The impact of DEO and its primary components, such as carvone, on the water-holding capacity (WHC) of fish muscle has been explored in various studies [8,69]. These earlier investigations highlighted the potential of DEO to enhance WHC through its antioxidant properties and interactions with muscle proteins. On the third day post-treatment, DEO-2-treated fish exhibited the highest WHC value compared to all other groups, including the control. DEO-2 likely contains an optimal concentration of bioactive compounds, such as carvone and limonene, which stabilize muscle proteins and enhance their ability to retain moisture. These compounds may interact with myofibrillar proteins like actin and myosin, reducing proteolytic degradation and maintaining the integrity of the muscle structure. Additionally, DEO-2’s antioxidative properties may prevent lipid oxidation, which can lead to the formation of hydrophobic products that reduce WHC [69]. From day 6 onward, all treated groups (BHT, DEO-1, DEO-2, and DEO-3) exhibited similar WHC values compared to the control group. The control group showed a significant decrease in WHC by the end of the storage period (day 15), indicating progressive dehydration due to proteolytic activity and oxidative damage [70]. Endogenous enzymes like calpains and cathepsins degrade myofibrillar proteins, leading to increased water release and reduced WHC [71]. Regarding purge loss (PL), the current findings revealed significant differences in PL trends among the groups, particularly at specific time points. The interplay between WHC and PL is driven by protein oxidation, proteolysis, and structural modifications in muscle fibers. As storage progresses, oxidative stress leads to carbonylation and aggregation of myofibrillar proteins, reducing their ability to retain water and causing increased PL [72]. Essential oil components, such as carvone and limonene, have been reported to mitigate these effects by scavenging free radicals and inhibiting pro-oxidant metal ions, thereby stabilizing protein structure and maintaining hydration capacity [73]. Cooking loss (CL) is a critical parameter that reflects the ability of muscle proteins to retain water during heat processing. It is influenced by factors such as protein denaturation, oxidative modifications, and muscle structure integrity. The results indicate that while the BHT and DEO treatments did not significantly affect CL throughout most of the storage period (*p* > 0.05), certain differences emerged at specific time points. On day 12, fish treated with BHT exhibited significantly higher CL compared to all other groups (*p* < 0.05). This increase in CL suggests that lipid and protein oxidation may have compromised the muscle’s water-holding properties. Previous studies have reported that synthetic antioxidants like BHT primarily target lipid oxidation but may have limited effects on protein oxidation, which is a key factor influencing WHC and, consequently, CL [72]. In contrast, DEO-1 and DEO-2 exhibited distinct trends, with DEO-2 showing superior late-stage stability. These results underscore the potential of DEO as a natural alternative for preserving water retention properties in stored fish meat, particularly in mitigating protein oxidation-related cooking losses. Oxidative modifications can lead to protein cross-linking, aggregation, and reduced WHC, resulting in higher CL [74].

The scatter plot matrix provides insights into the effects of storage time and treatment formulations on meat physicochemical properties. The correlations suggest that acidity alone does not significantly influence the water retention capacity of stored meat. Instead, other physicochemical and structural factors, such as protein oxidation, myofibrillar interactions, and lipid stability, likely play a more dominant role in determining WHC. This observation aligns with previous findings, indicating that protein denaturation and oxidative modifications can impact the ability of muscle fibers to retain water, rather than pH being the sole determinant. The results indicate that DEO-2 and DEO-3 exhibit potential benefits in maintaining WHC and reducing CL, which could contribute to improved meat quality during extended storage. The observed trends in pH, WHC, and PL suggest that oxidative and protein degradation processes are key determinants of quality deterioration over time. For food scientists and industry professionals, these findings underscore the importance of incorporating targeted antioxidant and protein-stabilizing interventions in meat preservation strategies. Future research should include controlled oxidative stability tests, rheological assessments, and sensory evaluations to further validate the efficacy of these treatments. Additionally, future research should focus on elucidating the molecular mechanisms underlying protein–water interactions and their implications for texture, juiciness, and overall consumer acceptability of stored and processed meat products.

The observed variations in the Warner–Bratzler Shear Force (WBSF) values among the different treatment groups can be attributed to the interplay between post-mortem proteolytic activities and the antioxidative properties of the applied treatments. In the control group, the progressive decline in WBSF values over time was indicative of muscle softening, primarily due to endogenous proteolytic enzymes such as calpains and cathepsins degrading myofibrillar proteins. This degradation leads to the weakening of the muscle structure, resulting in decreased shear force measurements [75,76]. By contrast, the BHT-treated group maintained higher WBSF values throughout the storage period. BHT, a synthetic antioxidant, inhibits lipid oxidation and the subsequent oxidative damage to muscle proteins. BHT preserves the integrity of myofibrillar structures by preventing the formation of ROS, thereby mitigating the extent of proteolysis and maintaining muscle firmness [77,78]. The DEO-treated group exhibited an initial increase in WBSF values followed by a decline. This pattern suggests that DEO may initially enhance muscle firmness, possibly through the stabilization of muscle proteins by its antioxidative components. The initial increase in WBSF in the DEO-treated group could be attributed to several mechanisms, including the presence of bioactive compounds, such as carvone, limonene, and other terpenoids, which are known for their antioxidant properties. These compounds can scavenge reactive oxygen species (ROS) and prevent oxidative damage to myofibrillar proteins such as actin and myosin, which are critical for maintaining muscle texture [8,79]. Certain terpenes in DEO have been shown to form hydrogen bonds or hydrophobic interactions with myofibrillar proteins, potentially stabilizing their conformations [8,80,81]. However, direct evidence linking DEO components to the promotion of cross-linking in myofibrillar proteins is limited, and studies on similar natural compounds, particularly polyphenols, provide relevant insights [82,83]. These interactions and cross-linking interactions between DEO components and muscle proteins can increase the resistance of muscle fibers to shear forces, resulting in higher WBSF values, which are indicative of tougher meat texture in the initial storage period. However, over time, the protective effect diminishes due to the degradation of DEO bioactive compounds during storage, leading to increased proteolytic activity and subsequent muscle softening [8,84,85].

The color parameters—L*, a*, b*—and color stability parameters such as Hue, Chroma, and Delta E (∆E) are crucial indicators of fish quality and shelf-life stability. The lightness parameter (L*) reflects the brightness or whiteness of the fish muscle, which is an important indicator of freshness and consumer appeal. On Day Zero, fish treated with the highest concentration of Dill essential oil (DEO-3) exhibited the lowest L* values, indicating reduced lightness. This initial decrease in lightness was also noticed with the application of Dill seed essential oil (DSEO) in meat products and was attributed to interactions of DEO’s bioactive compounds (such as carvone and limonene), which present with the highest concentration in DEO-3 than other levels, with muscle pigments or lipids [8]. In contrast, the BHT and other DEO treatments preserved lightness better, likely due to their antioxidative properties that prevent pigment degradation. By day 3, all treated groups exhibited higher L* values than the control, indicating that these treatments effectively slowed down oxidative processes that generated darker meat. DEO-2 was particularly effective at this stage [86]. On day 9, DEO-2-treated fish showed the lowest L*, suggesting some degree of browning or discoloration, while other groups were not significantly different from the control. This could be attributed to the transient nature of DEO-2’s antioxidative effects or interactions with muscle pigments or the enzymatic breakdown of DEO active components [8]. By day 12, all treated groups maintained higher L* values than the control, highlighting their ability to preserve lightness during mid-storage. On day 15, the sharp decrease in L* for BHT-treated fish was perhaps attributable to the onset of spoilage, advanced protein oxidation, and pigment degradation, likely due to the restricted antioxidant activity of BHT to inhibit lipid oxidation. DEO-2-treated fish maintained higher L* values, suggesting sustained antioxidative effects and better lightness preservation [8]. The redness index (a*) measures the intensity of red color in fish muscle, primarily influenced by myoglobin stability and oxidation. On Day Zero, no treatment significantly affected redness, indicating that myoglobin stability was not immediately impacted by the treatments [87]. By days 3–6, the BHT-treated fish showed lower a* values, possibly due to reduced myoglobin stabilization [8]. In contrast, the DEO-1-treated fish exhibited higher a* values, suggesting enhanced preservation of red pigments. On day 6, the DEO-3-treated fish displayed the highest a* values, likely due to the highest level of antioxidant components that might generate higher myoglobin protection than other levels [88]. By day 9, all treated groups, particularly DEO-3, exhibited higher a* values than the control, confirming better preservation of red color. This suggests that DEO treatments may inhibit oxidative reactions that degrade myoglobin. The increased redness in the treated groups indicates that the antioxidative effects of DEO treatments were more pronounced, preventing the oxidation of myoglobin and maintaining the red color. Carvone, the major component of DEO may chelate pro-oxidant metal ions, such as iron and copper [73]. The iron and copper ions are responsible for catalyzing oxidative reactions leading to myoglobin oxidation [89]. Therefore, by binding these metals, carvone can inhibit their oxidative activity, thereby preserving myoglobin in its reduced, oxygen-binding state. On days 12–15, redness increased in all groups, with BHT- and DEO-1-treated fish reaching their highest values. The increased redness in untreated fish could be due to oxidation-induced changes in myoglobin, leading to altered color perception. The treated groups’ higher a* values at later stages suggest that their antioxidative effects became more apparent over time, stabilizing and enhancing redness. The yellowness index (b*) reflects the intensity of yellow tones in fish muscle, often influenced by lipid oxidation and pigment interactions. On Day Zero, BHT- and DEO-treated fish (except DEO-3) showed higher b* values, indicating initial yellowing effects. This could be attributable to the DEO contents of yellowish pigments such as carotenoids and their potential isomers [90,91] or the formation of yellowish oxidation products [92]. The higher contents of DEO of yellow pigments such as carotenoids and their potential isomers, which simultaneously had strong antioxidant actions, clearly contributed to sustained higher yellowness values in DEO-2- and DEO-3-treated fish until the ninth storage day compared to other groups. On day 12, the sharp decrease in b* values for most treated groups suggests that antioxidative processes slowed down or stabilized after an initial peak [67]. By day 15, all treated groups exhibited higher b* values than the control. This observation may be the result of the deterioration of DEO natural yellow pigment with extended storage, loss of antioxidant effectiveness, and subsequent production of yellowish oxidation products [92].

The native aerobic plate count (APC) microorganisms were suppressed by higher concentrations of DEO, particularly DEO-3, maintaining microbial levels below critical thresholds until the ninth day, while the control samples exceeded these limits earlier. Similar inhibitory effects were observed for *lactic acid bacteria* (*LAB*) and coliforms, with Dill oil treatments, especially DEO-3, showing notable antimicrobial activity by decelerating microbial proliferation. On day 9, the staphylococcal counts were noticeably lower in the DEO-treated fillets than in the control samples. The current noticeable DEO antimicrobial properties are aligned with the findings reported on the microbiological quality of beef burgers [8]. The strong antimicrobial efficacy of DEO can be attributed to the high contents of bioactive components and their specific modes of action. The antimicrobial efficacy of DEO in suppressing spoilage-causing microorganisms is evident. Dill oil contains a high concentration of biologically active substances, primarily terpenes (such as carvone, limonene, and myrcene), phenolic compounds, and flavonoids [8]. These components are recognized for their ability to combat microbes, effectively targeting both Gram-positive and Gram-negative bacterial strains [93]. These compounds disrupt the microbial cell membranes, leading to increased permeability and subsequent cell death [94]. Additionally, bioactive components interfere with bacterial enzyme systems, thereby hindering essential metabolic pathways. Studies have demonstrated the effectiveness of DEO against various microorganisms, including *Aspergillus niger*, *Saccharomyces cerevisiae*, and *Candida albicans* [94]. Furthermore, research has indicated that Dill oil exhibits antibacterial activity against strains such as *Staphylococcus aureus*, *Escherichia coli*, and *Salmonella* Typhimurium. Current study findings support the potential application of Dill oil as a natural preservative in food products, enhancing safety and extending shelf life [95]. By day 9, the cumulative antimicrobial activity of DEO may have peaked, with sustained interactions with microbial cells leading to a noticeable reduction in staphylococcal counts [96]. At this point, spoilage microbes that were initially resistant may have been overwhelmed by the consistent antimicrobial pressure of DEO. The prolonged action of DEO likely caused irreparable damage to bacterial cells, reducing their ability to recover and proliferate, even under favorable conditions, which explains the lower count in the control on days 12 and 15.

The sensory evaluation results highlight the effectiveness of DEO treatments, particularly at higher concentrations (DEO-3), in preserving the quality attributes of fish meat during storage. Several studies support the efficacy of DEO in preserving the sensory qualities of fish meat during storage [21,97]. For instance, research has demonstrated that DEO exhibits significant antimicrobial and antioxidant activities, which contribute to maintaining the chemical and sensory properties of minced meat over an 18-day storage period [98]. These findings suggest that DEO, particularly at optimal concentrations, can serve as a natural alternative to synthetic antioxidants like BHT, offering improved sensory preservation and extended shelf life for fish meat.

The findings of this study have promising practical implications for the meat processing industry. The demonstrated efficacy of DEO in maintaining meat quality—by reducing purge and cooking losses, preserving pH, and improving tenderness during storage—suggests its potential use as a natural alternative to synthetic antioxidants such as BHT. With increasing consumer demand for clean-label and natural food products, incorporating DEO into meat preservation protocols could offer a safe, effective, and sustainable solution. Furthermore, its antimicrobial and antioxidant properties could reduce reliance on artificial preservatives, enhancing both product shelf life and consumer appeal in retail environments.

## 5. Conclusions

This study investigated the preservative effects of *Anethum graveolens* essential oil (DEO) on the quality and shelf life of Basa fish fillets during refrigerated storage. GC-MS analysis revealed the presence of monoterpenes such as α-phellandrene, d-limonene, carvone, and Dill ether in DEO. DEO exhibited dose-dependent antioxidant activity and antibacterial efficacy against various foodborne pathogens. Fish fillets treated with DEO, particularly at higher concentrations, effectively maintained pH, water-holding capacity, and color stability compared to the control. Microbial analysis showed that DEO significantly reduced the growth of aerobic plate count, lactic acid bacteria, coliforms, and Staphylococci. Sensory evaluation indicated that DEO treatments preserved the odor, color, texture, and overall acceptability of the fish fillets throughout the storage period. These findings demonstrate the potential of *Anethum graveolens* essential oil as a natural preservative to enhance the quality and extend the shelf life of fish fillets during refrigerated storage. Finally, the current study focused on only one type of fish and one constant storage setting; such constraints may be considered in future investigations to clarify the consequences of DEO preservative traits. Future research should also consider defining mechanisms of action and estimating in vivo safety data.

## Figures and Tables

**Table 1:** Chemical composition of Dill essential Oil.

No.	Compound	Chemical Family	RT	RI	Area, %
1	2-thujene	Monoterpene	4.71	915	0.32
2	β-ocimene	Monoterpene	4.84	944	1.84
3	Sabinene	Monoterpene	5.60	820	0.17
4	α-pinene	Monoterpene	6.04	886	0.36
5	β-pinene	Monoterpene	6.04	878	0.36
6	α-phellandrene	Monoterpene	6.33	938	21.81
7	p-cymene	Monoterpene	6.71	936	8.89
8	d-limonene	Monoterpene	6.93	941	18.54
9	Dill ether	Monoterpene	10.83	947	14.82
10	Camphor	Monoterpene	11.10	911	0.93
11	Camphene	Monoterpene	11.33	883	0.61
12	Carvone	Monoterpene	12.21	933	17.42
13	Terpineol	Monoterpene	12.48	868	1.21
14	Carvyl acetate	Monoterpene	14.69	779	1.73
15	Isodillapiole	phenylpropanoid	16.38	700	0.58
16	α-sinensal	Sesquiterpenoid	26.77	810	0.23
17	Apiol	Phenylpropene	21.72	879	10.30

## Data Availability

The original contributions presented in this study are included in the article. Further inquiries can be directed to the corresponding author.
